# Treatment and control of modifiable cardiovascular risk factors among patients with diabetes mellitus and hypertension in Inner Mongolia: A cross‐sectional study

**DOI:** 10.1111/jch.14375

**Published:** 2021-10-26

**Authors:** Yanqing Bi, Zixuan Tian, Wenyan Yan, Min Liu, Yuqian Zhao, Han Bao, Tao Yan, Nan Zhang, Yuan Xia, Xingguang Zhang

**Affiliations:** ^1^ Department of Health Statistics Public Health College Inner Mongolia Medical University Hohhot PR China

**Keywords:** cardiovascular disease, control, diabetes, hypertension, Inner Mongolia, treatment

## Abstract

The authors assessed treatment and control of blood glucose, blood pressure (BP), and blood lipids among patients from Inner Mongolia with diabetes mellitus (DM) and hypertension (HTN) and identified the modifiable factors associated with treatment and achievement of blood glucose, BP, and blood lipid targets. The authors used a multistage stratified cluster sampling method according to geographical location and level of economic development in Inner Mongolia. Among patients with DM and HTN, the crude rates of fasting plasma glucose (FPG) treatment and control was 30.76% and 4.73%, respectively. Crude rates of BP treatment and control were 50.81% and 8.70%, respectively. The authors found that treatment rates of HTN and DM and control rates of BP and FPG showed a gradually increasing trend with increased age. Among patients with DM and HTN, the likelihood of treatment for HTN and DM was significantly increased among participants who were older, non‐Mongolian, male, obese, smokers, and those with previous cardiovascular disease. The authors found that control of BP, FPG, and low‐density lipoprotein cholesterol was far from optimal among study participants. Medical and health departments in Inner Mongolia should take appropriate measures to reduce the burden of DM and HTN in the population, such as by promoting and improving the quality of HTN and DM treatment to achieve control goals and reduce the risk of cardiovascular disease.

## 1 INTRODUCTION

Type 2 diabetes mellitus (DM) and hypertension (HTN) are established risk factors for cardiovascular disease (CVD).^1^, ^2^, ^3^, ^4^ Moreover, patients with DM and HTN have an increased risk of cardiovascular mortality compared with patients who have either condition alone.^5^, ^6^ Patients with HTN and DM have a higher risk of heart disease or stroke.^3^, ^4^, ^7^ Further, DM and HTN are common conditions that frequently present together. One study reported that up to 75% of adults with DM also have HTN, and patients with HTN alone often show evidence of insulin resistance.^8^ HTN and DM are common, intertwined conditions with considerable overlap with respect to underlying risk factors.^9^

The current epidemic of DM and HTN has become a serious public health issue worldwide. Particularly in developing countries, a substantial gap exists between clinical practice and recommended treatment in the management of patients with DM and HTN. With changes in lifestyles and a growing aging population, the prevalence of DM and HTN among individuals in China has increased rapidly over the past decades. Considerable uncertainty remains about the prevalence of DM and HTN according to age, sex, region, and socioeconomic status, as well as current levels of blood pressure (BP), blood glucose, and blood lipid management among patients in Inner Mongolia. Limited evidence is available on the prevalence and management of patients with DM and HTN in this region. In the present study, we aimed to assess the treatment and control of blood glucose, BP, and blood lipids among patients with patients with DM and HTN in Inner Mongolia and to explore those factors associated with treatment and achievement of blood glucose, BP, and blood lipid target goals. We also provide an effective scientific basis for the prevention and control of CVD and death in high‐risk groups.

## 2 METHODS

### 2.1 Study design and participants

The data in this study were from the China Patient‐Centered Evaluative Assessment of Cardiac Events Million Persons Project in Inner Mongolia. We used a multistage stratified cluster sampling method in participant selection, conducted during 2015–2017. According to geographic location and economic development level, six cities were randomly selected from the Inner Mongolia Autonomous Region: Hohhot, Wuhai, Chifeng, Xing'an, Ordos, and Hulun Buir. In the second stage, we selected one district or county from each of these cities according to the size of the district or county and population stability. Finally, potential participants from each community or village were invited to participate in the study by local staff through an extensive publicity campaign on television, radio, and in newspapers.

This study was approved by the Central Ethics Committee of the National Cardiovascular Center of China. All participants provided written informed consent. We recruited residents aged 35–75 years who had lived in Inner Mongolia for at least 1 year. Finally, a total of 70 380 participants from Inner Mongolia were registered. Among them, 9617 patients with DM and HTN were included in this study.

### 2.2 Data collection

We used trained investigators as well as checks of participants' responses by trained interviewers to ensure the validity of the self‐reported data. We collected basic information of sociodemographic characteristics such as age, sex, ethnic group, education level, annual household income, smoking status, history of disease, and living environment. Data of height, weight, BP, fasting blood glucose (FPG), and blood lipids were obtained in physical examinations or laboratory testing.

BP was measured twice by a medical staff member who had received professional training, using an electronic blood pressure monitor (Omron HEM‐7430; Omron Corporation, Kyoto, Japan) and a standard protocol. One of two cuff sizes (regular adult or large) was chosen according to arm circumference and the procedure was conducted by a trained, certified observer who used an American Heart Association protocol to perform three BP measurements after the participant had been at rest in a seated position for at least 5 min. The mean of the two recorded values of systolic blood pressure (SBP) and diastolic blood pressure (DBP) was used in all analyses. All devices were regularly maintained and calibrated to ensure consistency of the measurements. After at least 12 h of overnight fasting, venous blood samples were collected for the measurement of FPG and lipid. Blood glucose was measured using a glucose analyzer (BeneCheck PD‐G001‐2, Taiwan, China). Blood lipid tests were performed to measure total cholesterol (TC), triglyceride (TG), high‐density lipoprotein cholesterol (HDL‐C), and low‐density lipoprotein cholesterol (LDL‐C) using a rapid lipid analyzer (CardioChek PA Analyzer; Polymer Technology Systems, Indianapolis, IN, USA).

### 2.3 Definition of variables

Current smokers were defined as having smoked at least one cigarette a day during the past year. Body mass index (BMI) was calculated as weight (in kilograms) divided by height (in meters) squared. According to recommendations of the Working Group on Obesity in China, obesity was defined as BMI ≥28 kg/m^2^. Mongol was the main minority ethnic group in this study. The ethnicity of participants was categorized into three groups: Han, Mongol, and other minority ethnic groups. Participants with either TC ≥6.22 mmol/L, LDL‐C ≥4.14 mmol/L, TG ≥2.26 mmol/L, HDL‐C < 1.04 mmol/L, or self‐reported use of lipid‐lowering drugs were classified as having dyslipidemia.^10^ A history of CVD was defined as any self‐reported history of myocardial infarction, stroke (including hemorrhagic stroke and ischemic stroke), percutaneous coronary intervention, or coronary artery bypass grafting. Risk factors for cardiovascular disease included underlying diseases such as hypertension, diabetes, obesity, and hypercholesterolemia.

Patients with DM were defined as those who regularly took hypoglycemic drugs, received insulin injections, or had an FPG > 7.0 mmol/L or self‐reported diabetes. HTN was defined as SBP ≥140 mm Hg or DBP ≥90 mm Hg or use of antihypertensive medication within the previous 2 weeks (in accordance with 2010 Chinese guidelines for the management of hypertension^11^). Among patients with DM and HTN, those who self‐reported taking at least one antihypertensive medication were considered to be under treatment for HTN, and patients with SBP ≤140 mm Hg and DBP ≤90 mm Hg were considered to have achieved the treatment target of BP.^12^ Patients who reported regular use of anti‐diabetic drugs or insulin injections within a 2‐week period were considered to have received diabetes treatment. Patients with FPG > 4.4 mmol/L and < 7.0 mmol/L were considered to have achieved the treatment goal of diabetes. Similarly, among patients with dyslipidemia, those who reported taking one or more lipid‐lowering drugs were considered to be receiving treatment for dyslipidemia. Among patients with dyslipidemia, those without CVD who had LDL‐C < 2.6 mmol/L and those with prior CVD who had LDL‐C < 1.8 mmol/L were considered to have reached the treatment target of LDL‐C.

### 2.4 Statistical analysis

Continuous variables are presented as mean and standard deviation (SD) or median and compared using the chi‐square test. Categorical variables are shown as number and percentage. We calculated the rates of both treatment and control of BP, FPG, and LDL‐C according to the different subgroups, as well as 95% confidence intervals (CIs). Age‐ and sex‐standardized rates of treatment and control for diabetes and HTN at national level were calculated using a direct method of standardization^13^ and the standard population from the 2010 Chinese census.

To analyze the association between individual characteristics and treatment as well as control of DM and HTN, multivariate logistic models were developed with stepwise selection of independent variables. In the multivariate analysis, the independent variables included age group (35–45, 46–55, 56–65, and 66–75 years), sex (female/male), married status (yes or no), obesity (yes or no), high school degree or above (yes or no), annual income ≥50,000 RMB (yes or no), farmer (yes or no), residence (urban or rural), ethnic group (Han, Mongol or other minority ethnic group), and history of cardiovascular disease (yes or no). All the analyses were conducted using SAS version 9.4 (SAS Institute, Inc., Cary, NC, USA). Two‐sided *p *< .05 was considered statistically significant.

## 3 RESULTS

A total of 9617 patients with DM and HTN were included in this study; the study flow chart is shown in Figure S1. Overall, 13.66% (9617/70380) of participants had DM and HTN. Characteristics of the study population are shown in Table 1.

**TABLE 1:** Baseline characteristics of the study population

Characteristics	Patients with HTN and DM (*N* = 9617)
Age(years, mean±SD)	57.72±8.41
Age group, *n* (%)	
35–45	850(8.84)
46–55	2829(29.42)
56–65	4155(43.20)
66–75	1783(18.54)
Sex, *n* (%)	
Female	5342(55.55)
Male	4275(44.45)
Ethnic group, *n*(%)	
Han	8674(90.19)
Mongol	774(8.05)
Other minority	169(1.76)
Education, *n* (%) High school or above	
Yes	7011(70.90)
No	2606(27.10)
Annual household income≥50000, *n*(%)	
Yes	1113(11.57)
No	8504(88.43)
Farmer, *n*(%)	
Yes	4080(42.42)
No	5537(57.58)
Location of residence, *n*(%)	
Rural	6266(65.16)
Urban	3351(34.84)
Marital status, *n*(%)	
Married	8543(88.33)
Not married	1074(11.17)
Current smoker, *n*(%)	
Yes	2213(23.01)
No	7404(76.99)
BMI, kg/m^2^	27.14±3.42
Obesity	
Yes	3673 (38.19)
No	5944 (61.81)
FPG, mmol/L	8.65±2.28
SBP, mm Hg	155.45±18.25
DBP, mm Hg	90.53±11.03
TC, mmol/L	4.79±1.09
LDL‐C, mmol/L	2.54±0.86
TG, mmol/L	1.68(1.19‐2.50)
HDL‐C, mmol/L	1.33±0.39

Data are shown as mean SD, median (interquartile range), or *n* (%).

*Abbreviations*: BMI, body mass index; DBP, diastolic blood pressure; DM, diabetes mellitus; FPG, fasting blood glucose; HDL‐C, high‐density lipoprotein cholesterol.; HTN, hypertension; LDL‐C, low‐density lipoprotein cholesterol; SBP, systolic blood pressure; SD, standardized deviation; TC, total cholesterol; TG, triglyceride.

### 3.1 Treatment and control of FPG, BP, and LDL‐C

The treatment and control of FPG, BP, and LDL‐C by different groups are displayed in Tables 2 and 3. The standardized treatment rate and control rate of DM, HTN, and dyslipidemia were slightly lower than the crude rate (Tables 2 and 3).

**TABLE 2:** Treatment rates for DM, HTN and dyslipidemia in the study population stratified by individual characteristics

	Treatment of DM% (95% CI)^a^	Treatment of HTN% (95% CI)^a^	Treatment of dyslipidemia% (95% CI)^a^
Overall crude rates	31.59(30.66–32.51)	50.81(49.82–51.81)	5.01(4.58–5.45)
Standardized rates^b^	25.36(25.35–25.36)	42.83(42.83–42.84)	3.92(3.91–3.92)
Age group			
35–45	16.59(14.08–19.09)	31.18(28.06–34.30)	1.65(0.79–2.50)
46–55	27.08(25.44–28.71)	46.98 (45.14–48.82)	4.28(3.53–5.02)
56–65	34.90(33.45–36.35)	53.67(52.15–55.19)	5.92(5.20–6.64)
66–75	38.19(35.94–40.45)	59.62(57.34–61.90)	5.67(4.59–6.74)
Sex			
Female	29.47(28.10–30.94)	46.38(44.89–47.88)	4.63(4.00–5.26)
Male	33.28(32.01–34.54)	54.36(53.02–55.69)	5.32(4.71–5.92)
Ethnic group			
Han	32.39(31.41–33.38)	51.54(50.49–52.59)	5.12(4.65–5.58)
Mongol	22.60(19.65–25.56)	43.66(40.16–47.17)	3.88(2.51–5.24)
Other minority ethnic	31.36(24.29–38.42)	46.15(38.56–53.74)	4.73(1.50–7.97)
Education (high school degree or above)			
Yes	33.15(31.34–34.96)	49.34(47.42–51.26)	4.68(3.87–5.49)
No	31.00(29.92–32.09)	51.36(50.19–52.53)	5.13(4.62–5.65)
Annual household income(≥50000)			
Yes	36.56(33.73–39.40)	50.49(47.55–53.43)	5.21(3.90–6.52)
No	30.93(29.95–31.92)	50.85(49.79–51.92)	4.99(4.52–5.45)
Residence			
Urban	36.40(34.77–38.03)	50.79(49.55–52.03)	5.43(4.66–6.20)
Rural	29.01(27.88–30.13)	50.85(49.15–52.54)	4.79(4.26–5.32)
Marital status			
Married	32.03(31.04–33.02)	50.90(49.84–51.96)	4.97(4.51–5.44)
Not married	28.02(25.33–30.71)	50.09(47.09–53.08)	5.31(3.97–6.65)
Farmer			
Yes	27.03(25.67–28.39)	49.87(48.34–51.41)	4.90(4.24–5.56)
No	34.94(33.69–36.20)	51.50(50.19–52.82)	5.09(4.51–5.67)
Current smoker			
Yes	29.55(27.65–31.45)	48.35(46.26–50.43)	5.15(4.23–6.07)
No	32.19(31.13–33.26)	51.55(50.41–52.69)	4.97(4.48–5.47)
Obesity			
Yes	31.39(29.88–32.89)	57.11(55.51–58.72)	5.34(4.61–6.06)
No	31.71(30.52–32.89)	46.92(45.65–48.19)	4.81(4.27–5.36)
Prior CVD			
Yes	38.04(34.67–41.42)	65.08(61.76–68.39)	15.52(13.00–18.04)
No	31.00(30.03–31.97)	49.52(48.47–50.56)	4.06(3.65–4.47)

*Abbreviations*: CI, confidence interval; CVD, cardiovascular disease.; DM, diabetes mellitus; HTN, hypertension.

^a^ The value is presented in percent (95% confidence interval).

^b^ Age‐and sex‐standardized rates at national level.

**TABLE 3:** Controlled rates of FPG, BP, and LDL‐C in the study population stratified by individual characteristics

Characteristics	Control of FPG% (95% CI)^a^	Control of BP% (95% CI)^a^	Control of LDL‐C% (95% CI)^a^
Overall crude rates	5.13(4.69–5.57)	8.70(8.14–9.27)	56.44(55.45–57.43)
Standardized rates^b^	3.97(3.97–3.98)	7.93(7.93–7.94)	52.94(52.94–52.95)
Age group			
35–45	2.00(1.06–2.94)	5.88(4.30–7.47)	69.88(66.79–72.97)
46–55	4.14(3.40–4.87)	9.40(8.33–10.48)	57.83(56.01–59.65)
56–65	5.75(5.04–6.46)	10.30(9.38–11.23)	53.89(52.37–55.40)
66–75	6.73(5.57–7.89)	10.32(8.90–11.73)	53.79(51.47–56.10)
Sex			
Female	4.66(4.02–5.29)	9.33(8.46–10.21)	51.50(50.16–52.84)
Male	5.50(4.89–6.11)	9.90(9.10–10.70)	62.62(61.17–64.07)
Ethnic group			
Han	5.30(4.83–5.77)	10.07(9.44–10.70)	56.58(55.54–57.63)
Mongol	3.62(2.30–4.94)	5.43(3.82–7.02)	55.36(52.17–57.16)
Other minority ethnic	2.96(0.37–5.53)	7.10(3.19–11.01)	55.68(52.18–59.19)
Education (high school degree or above)			
Yes	5.25(4.40–6.11)	11.36(10.13–12.58)	57.48(55.58–59.38)
No	5.08(4.56–5.59)	9.01(8.34–9.68)	56.05(54.89–57.22)
Annual household income(≥50000)			
Yes	6.38(4.94–7.82)	9.70(7.96–11.45)	56.78(53.87–59.69)
No	4.96(4.50–5.42)	9.64(9.01–10.27)	56.40(55.34–57.45)
Residence			
Urban	6.86(6.00–7.72)	9.26(8.32–10.20)	51.66(49.96–53.35)
Rural	4.20(3.70–4.69)	9.42(8.69–10.14)	59.00(57.78–60.22)
Marital status			
Married	5.22(4.75–5.69)	10.04(9.41–10.68)	57.18(56.13–58.23)
Not married	4.38(3.15–5.60)	6.52(5.04–8.00)	50.56(47.56–53.55)
Farmer			
Yes	4.34(3.71–4.96)	8.55(7.70–9.41)	58.73(57.21–60.24)
No	5.71(5.10–6.31)	10.46(9.65–11.26)	54.76(53.45–56.07)
Current smoker			
Yes	4.47(3.61–5.34)	10.98(9.68–12.28)	62.68(60.66–64.69)
No	5.32(4.81–5.83)	9.25(8.59–9.91)	54.58(53.44–55.71)
Obesity			
Yes	4.49(3.82–5.16)	9.23(8.29–10.17)	56.60(55.00–58.21)
No	5.52(4.94–6.10)	9.91(9.14–10.67)	56.34(55.08–57.60)
Prior CVD			
Yes	7.76(5.90–9.62)	15.14(12.65–17.63)	23.40(20.46–26.35)
No	4.89(4.43–5.34)	9.15(8.55–9.75)	59.44(58.41–60.46)

*Abbreviations*: BP, blood pressure; CVD, cardiovascular disease; FPG, fasting blood glucose; LDL‐C, low‐density lipoprotein cholesterol.

^a^ The value is presented in percent (95% confidence interval).

^b^ Age‐ and sex‐standardized rates at national level.

We found that treatment rates for HTN and DM were higher in older patients (*p*_trend _< .05). Similarly, the control rates of BP and FPG also increased with age (*p*_trend _< .05). Ethnic Mongolian participants had lower rates of treatment and control for DM, HTN, and hyperlipidemia. The control rates of BP, blood glucose, and LDL‐C in married patients were significantly higher than those in unmarried patients (*p *< .05). Participants living in urban areas had higher rates of diabetes treatment and control. Furthermore, higher control rates of FPG, BP, and LDL‐C were found in the group with higher education levels. Compared with patients who did not have CVD, those with prior CVD had much higher rates of treatment and control for DM as well as HTN (*p *< .05).

### 3.2 Distance to treatment targets

**FIGURE 1:**
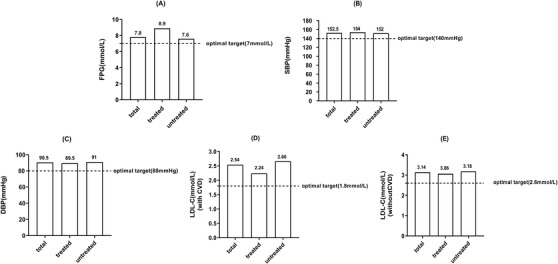
Difference in optimal treatment targets for FPG, BP, and LDL‐C among uncontrolled patients. Values presented as median and interquartile range (IQR). Dashed line represents the optimal treatment target

Differences in optimal treatment targets for FPG, BP, and LDL‐C were calculated in uncontrolled patients (Figure 1). We found that the difference in FPG and SBP optimal treatment targets was greater in treated than in untreated patients. The differences in optimal treatment target values of LDL‐C were smaller in treated patients with dyslipidemia.

The optimal treatment target distance to SBP was calculated as the measured value of SBP minus 140 (mm Hg) among patients with HTN and uncontrolled SBP. The optimal treatment target distance to DBP was calculated as the measured value of DBP minus 90 (mm Hg) among patients with HTN and uncontrolled DBP. The optimal treatment target distance to FPG was calculated as the measured value of FPG minus 7 (mmol/L) among patients with diabetes and FPG ≥7 (mmol/L). The optimal treatment target distance to the target of LDL‐C was calculated as the measured value of LDL‐C minus 1.8 (mmol/L) among patients with uncontrolled LDL‐C and prior CVD. In patients with uncontrolled LDL‐C and without prior CVD, the optimal treatment target distance was calculated as the measured value of LDL‐C minus 2.6 (mmol/L).

### 3.3 Factors influencing treatment and control of BP, FPG, and LDL‐C

**FIGURE 2:**
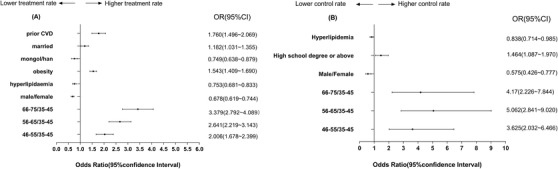
Odds ratios (ORs) for characteristics associated with treatment and control of hypertension in patients with hypertension and diabetes. (A) Treatment of hypertension; (B) achieving BP treatment target (< 140/90 mm Hg). CI, confidence interval; CVD, cardiovascular disease

**FIGURE 3:**
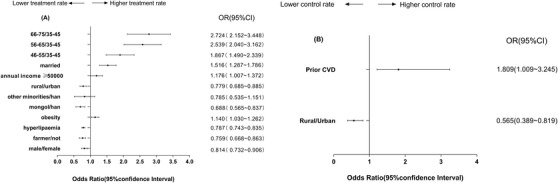
Odds ratios (ORs) for characteristics associated with treatment and control of diabetes in patients with hypertension and diabetes. (A) Treatment of diabetes; (B) achieving FPG treatment target (< 7.0 mmol/L). CI, confidence interval; CVD, cardiovascular disease; HTN, hypertension

**FIGURE 4:**
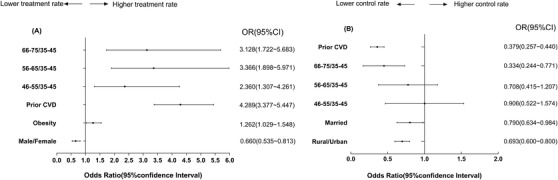
Odds ratios (ORs) for characteristics associated with treatment of dyslipidemia and control of LDL‐C in patients with hypertension and diabetes. (A) Treatment of dyslipidemia; (B) achieving LDL‐C treatment target (< 2.6 mmol/L for patients without CVD; < 1.8 mmol/L for patients with prior CVD). CI, confidence interval; CVD, cardiovascular disease; LDL‐C, low‐density lipoprotein cholesterol

Using a binary logistic model, we identified several independent influencing factors related to the treatment and control of patients with DM and HTN (Figures 2, 3, 4). Patients with prior CVD and those who were married, Han ethnicity, obese, female, or older had higher treatment levels. Patients without hyperlipidemia and those with higher education levels, female sex, and older age were more likely to achieve BP control goals (Figure 2).

Participants with Mongol ethnicity had lower odds of being treated for DM compared with those of Han ethnicity (Figure 3). Among patients with DM and HTN, those who were older, female, married, obese, not farmers, and had an annual household income ≥50,000 RMB were significantly more likely to be treated for DM. Patients who had prior CVD and those who lived in an urban area had better FPG control (Figure 3).

Among patients with dyslipidemia, patients with prior CVD and those who were older, married, or living in rural areas had difficulty achieving LDL‐C control goals (Figure 4). According to results of the binary logistic model, we further identified all relevant influencing factors. The results are shown in Table 1.

## 4 DISCUSSION

This study was the largest cross‐sectional investigation related to CVD, DM, and HTN in the Inner Mongolia Autonomous Region in recent years. This study, which included a large study population from multiple regions and comprising different minority ethnic groups, provides an up‐to‐date assessment of the treatment and control of FPG, BP, and LDL‐C among patients with DM and HTN in Inner Mongolia and identifies predictive factors for poor FPG, BP, and LDL‐C control. We are confident that the results of our research will help improve public health policies and effectively improve the level of public health in the region.

International clinical guidelines strongly recommend that optimal control levels of FPG, BP, and LDL‐C are important to prevent the risk of developing CVD in patients with DM combined with HTN.^14^, ^15^ However, we found that control levels of FPG (5.13%), BP (8.7%), and LDL‐C (56.44%) in our study population were far from ideal. We also found that high treatment rates do not mean that these risk factors are well prevented and controlled. Therefore, the health care sector should adopt relevant strategies to improve the quality and effectiveness of treatment to achieve control goals.

Whereas previous reports have shown that the prevalence of HTN among individuals in China is slightly lower, on average, than that in Western countries, this study showed that the prevalence of HTN in China is now comparable to that in the West.^16^ However, the level of diagnosis, treatment, and control of HTN in China is much lower than that in many high‐income countries.^17^, ^18^ Recent surveys show that more than two‐thirds of patients(England, the USA, and Canada) with HTN have been diagnosed, more than two‐thirds of those diagnosed have been treated, and approximately two‐thirds of patients have their BP under control.^17^ Compared with studies from developed countries, we observed lower control rates of FPG, BP, and LDL‐C in this study.^17^, ^19^, ^20^, ^21^ Low treatment rates of DM with HTN may be owing to poor awareness about the disease, low compliance with treatment, and inadequate health promotion information provided by the health sector.^22^ Moreover, national health authorities have not increased the attention paid to patients with DM and HTN, which is another major obstacle to achieving optimal control of BP, blood glucose, and LDL‐C.^23^

A series of studies have shown that in the management of patients with DM and HTN, comprehensive control of various risk factors that can aggravate the development of CVD can achieve good preventive effects.^21^, ^22^, ^24^ There is accumulating evidence that the rigorous treatment of HTN and other risk factors, such as dyslipidemia and hyperglycemia, considerably lessens the burden of CVD and renal disease in patients with DM and HTN.^25^ We found that the composite control rate of FPG, BP, and LDL‐C was 0.38% (37/9617). A nationwide, multicenter cross‐sectional survey in China reported that only 5.4% of patients with DM achieved all recommended targets for FPG, BP, and LDL‐C.^26^ Similarly, unsatisfactory composite control of risk factors has been reported in other developed countries.^20^ Therefore, it is an enormous challenge for China as well as other developed countries to achieve treatment targets for key indicators.

According to age group, we found that treatment and control of BP were lower in patients with HTN in the young and middle‐aged groups. The same trend was observed in patients treated with DM, which was consistent with the results of other studies.^27^, ^28^ Young patients lack awareness about health care, do not undergo regular physical examinations, and are not compliant with regular medication. A patient with early HTN and DM prior to the onset of complications and who is asymptomatic, in whom quality of life has not yet been affected, may not perceive the need for good BP and DM control.^29^ It was worth noting that the cure rate of dyslipidemia was low, but the control rate of LDL‐C is high. The defined indexes of dyslipidemia include TC, LDL‐C, TG, and HDL‐C. In the calculation process, the control of dyslipidemia was obtained only based on LDL‐C. In fact, one of the characteristics of dyslipidemia in the Chinese population is that the rate of abnormal TG levels is particularly high whereas the rate of abnormal LDL‐C levels is particularly low.^30^ Therefore, we found a high control rate for LDL‐C in this study. Additionally, we found urban–rural and ethnic differences in the treatment and/or control of FPG among groups according to residence and ethnicity. The treatment rate and control rates among Mongolian participants and other minority ethnic groups were lower than among participants with Han nationality. This suggests that greater attention should be paid to DM and HTN among minority ethnic groups and individuals living in rural areas.

On the whole, Inner Mongolia should improve its hierarchical diagnosis and treatment system for common diseases such as HTN and DM, optimize the allocation of health resources, and train health workers in other related aspects to contribute to the standardized management of chronic diseases and improve the utilization efficiency of primary health resources.

Our study had several limitations. First, we noted that 35% of BP measurement values ended in 0 or 5, which could indicate terminal digit preference among medical staff in recording BP measurements, despite the use of a standardized method. Second, using data for the total population of Inner Mongolia, we adjusted the main asymmetry characteristics of the research population, such as age, sex, and ethnicity. The adjusted estimates for the treatment and control of DM, HTN, and dyslipidemia were slightly lower than the crude estimates. Moreover, owing to sampling bias, we expect that the treatment rate and control rate were overestimated in our study because potential participants were more likely to yield positive results owing to greater concern about their health. This study was based on 2010 guidelines. In comparison with current, more stringent guidelines, the control rates based on 2020 guidelines were overestimated. Finally, only cross‐sectional estimates of BP, FPG, and LDL‐C control were counted and changes in these indicators could not be tracked.

## 5 CONCLUSIONS

In this study, we investigated prevalence, treatment, and control rates in a representative sample of middle‐aged and older Chinese patients with DM and HTN and found sex‐specific and age‐specific patterns. We found that control of BP and blood glucose was far from optimal in our sample. We also observed that high treatment rates do not mean that these risk factors are well controlled. Therefore, the health sector should adopt a variety of strategies to achieve control objectives, including promotion and improvement of the quality of treatment for DM and HTN.

## CONFLICT OF INTERESTS

The Authors declare that there is no conflict of interest.

## AUTHORS' CONTRIBUTIONS

Yanqing Bi and Xingguang Zhang contributed the central idea. Yanqing Bi wrote the initial draft of the paper. Wenyan Yan, Min Liu, Yuqian Zhao, Han Bao and Tao Yan analysed the data. The remaining authors contributed to refining the ideas, carrying out additional analyses and finalizing this paper. All authors gave final approval, and agreed to be accountable for all aspects of work ensuring integrity and accuracy (Yanqing Bi, Zixuan Tian, Wenyan Yan, Min Liu, Yuqian Zhao, Han Bao, Tao Yan, Nan Zhang, Xin Fang, Yuan Xia, Xingguang Zhang).

## Supporting information

Supporting information.
