# Supplementary material for: Treatment and control of modifiable cardiovascular risk factors among patients with diabetes mellitus and hypertension in Inner Mongolia: A cross‐sectional study
Source: J Clin Hypertens (Greenwich). 2021 Oct 26;23(11):2016–25. doi: 10.1111/jch.14375 (PMC8630609; doi:10.1111/jch.14375)
Supplement: Supplementary file 1 — Supporting information. [file JCH-23-2016-s001.pdf]

Supplemental Figure 1 Flowchart illustrating the study population

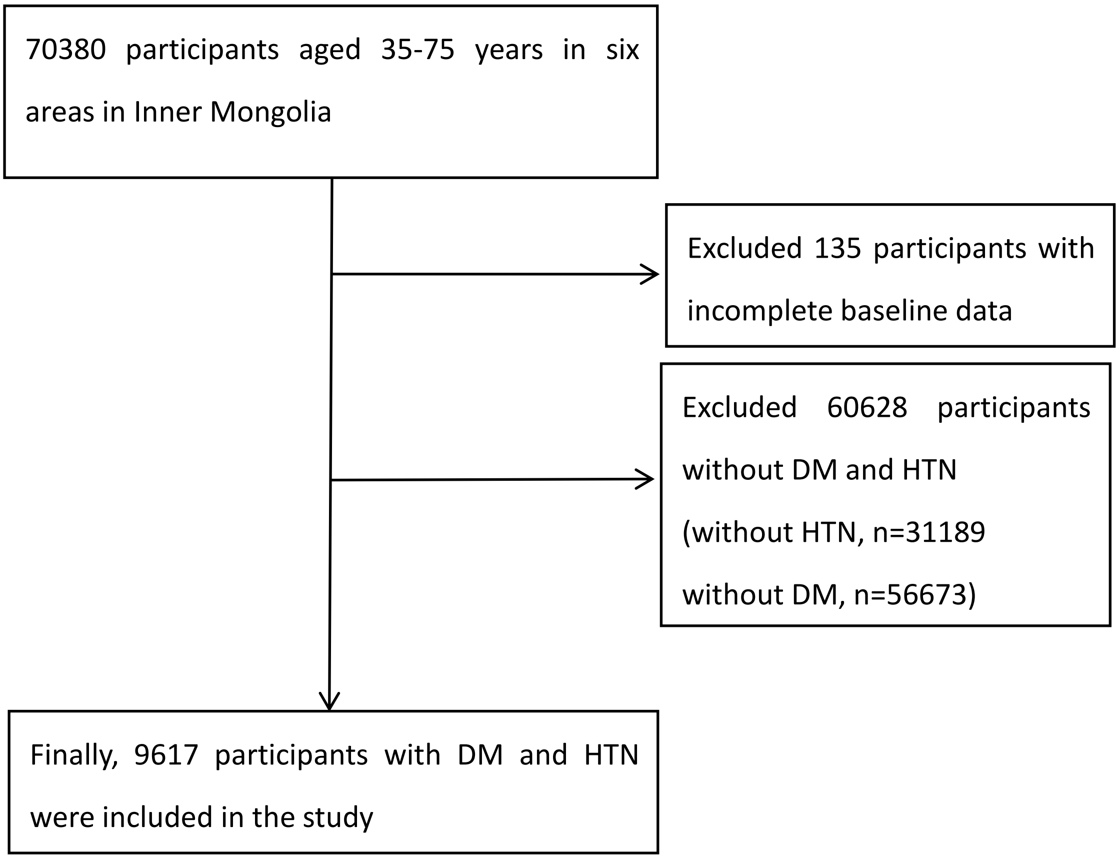

Abbreviations: HTN, Hypertension; DM, Diabetes mellitus;
